# Comparative Trends in Ischemic Heart Disease Admissions, Presentation and Outcomes Due to the COVID-19 Pandemic: First Insights From a Tertiary Medical Center in Pakistan

**DOI:** 10.7759/cureus.17558

**Published:** 2021-08-30

**Authors:** Ali Aahil A Noorali, Humza Thobani, Shiraz Hashmi, Sara Iqbal, Asma A Merchant, Mian Arsam Haroon, Sardar Shahmir B Chauhan, Saad Mallick, Nida Zahid, Yasir Khan, Osman Faheem, Saulat H Fatimi

**Affiliations:** 1 Medicine, Aga Khan University, Karachi, PAK; 2 Cardiothoracic Surgery, Aga Khan University, Karachi, PAK; 3 Education, Center for Innovation in Medical Education, Aga Khan University, Karachi, PAK; 4 Cardiology, Aga Khan University, Karachi, PAK

**Keywords:** developing countries, covid-19, coronary artery disease, myocardial ischemia, acute coronary syndrome

## Abstract

Introduction

COVID-19 has manifested a striking disarray in healthcare access and provision, particularly amongst patients presenting with life-threatening ischemic heart disease (IHD). The paucity of data from low-middle income countries has limited our understanding of the consequential burden in the developing world. We aim to compare volumes, presentations, management strategies, and outcomes of IHD amongst patients presenting in the same calendar months before and during the COVID-19 pandemic.

Methods

We conducted a retrospective cross-sectional analysis at the Aga Khan University Hospital, one of the premier tertiary care centres in Pakistan. Data were collected on all adult patients (>18 years) who were admitted with IHD (acute coronary syndrome (ACS) and stable angina) from March 1 to June 30, 2019 (pre-COVID) and March 1 to June 30, 2020 (during-COVID), respectively. Group differences for continuous variables were evaluated using student t-test or Mann-Whitney U test. The chi-squared test or Fisher test was used for categorical variables. Values of p less than 0.05 were considered statistically significant. P-value trend calculation and graphical visualization were done using STATA (StataCorp, College Station, TX).

Results

Data were assimilated on 1019 patients, with 706 (69.3%) and 313 (30.7%) patients presenting in each respective group (pre-COVID and during-COVID). Current smoking status (p=0.019), admission source (p<0.001), month of admission (p<0.001), proportions ACS (p<0.001), non-ST-elevation-myocardial-infarction (NSTEMI; p<0.001), unstable angina (p=0.025) and medical management (p=0.002) showed significant differences between the two groups, with a sharp decline in the during-COVID group. Monthly trend analysis of ACS patients showed the most significant differences in admissions (p=0.001), geographic region (intra-district vs intracity vs outside city) (p<0.001), time of admission (p=0.038), NSTEMI (p=0.002) and medical management (p=0.001).

Conclusion

These data showcase stark declines in ACS admissions, diagnostic procedures (angiography) and revascularization interventions (angioplasty and coronary artery bypass graft surgery, CABG) in a developing country where resources and research are already inadequate. This study paves the way for further investigations downstream on the short- and long-term consequences of untreated IHD and reluctance in health-seeking behaviour.

## Introduction

The COVID-19 pandemic, caused by the SARS-CoV-2 virus, has created considerable disarray in the access and provision of health services to other fields of medicine and surgery. Healthcare workers, medical resources and attention have all been diverted to cater to managing an exponential increase in COVID-19 infections, often at the cost of ignoring the pre-existing burden of communicable and non-communicable diseases [1,2]. This is particularly evident in resource-challenged countries with poorly developed healthcare infrastructure and limited avenues for crisis management in such situations - Pakistan being one of them [3].

The overwhelmingly acute burden of coronary artery disease (CAD) in low-middle income countries, LMICs, (13.63%) is even more pronounced in Pakistan (15.31%), with ischemic heart disease (IHD) being the largest cause of mortality in the country [4-6]. This problem is further aggravated by a burgeoning incidence of out-of-hospital cardiac arrests (OHCA) which, coupled with a lack of coordinated cardiac health services in most parts of the country, makes it near impossible to gauge the true burden of CAD and in Pakistan [7].

Interestingly, in spite of this overarching exponential increase in the burden of ischemic heart diseases, a curious trend has emerged during the COVID-19 pandemic. Multiple studies from across the world have consistently demonstrated a reduction in cases of the acute coronary syndrome (ACS), as well as both emergency and elective percutaneous coronary intervention (PCI) and coronary artery bypass graft (CABG) procedures [8-11]. However, there is a significant relative dearth of literature investigating or discussing these trends in LMICs. This is a critical gap in current knowledge, because while such studies may provide clarity to the CAD burden and management in high-income countries (HICs), it may not be appropriate to draw parallels to LMICs where the landscape of healthcare presents exacerbated challenges. Moreover, with radically different healthcare systems, staggeringly limited resources and differences in population behaviour with respect to their awareness and education regarding COVID-19, it is likely that observations stemming from research in HICs might not be applicable to LMICs such as Pakistan.

Exploring and following trends in cardiac surgeries is paramount to comprehend areas of deficiencies and plan interventions accordingly. Reporting data on trends in the volumes of PCI and CABG procedures being performed for ACS during the COVID-19 pandemic also bolsters cardiologists’ and cardiovascular surgeons’ ability to respond to the current and future pandemics. We aim to compare volumes, presentations, management strategies and outcomes amongst patients presenting from March 1 to June 30, 2019 (pre-COVID) and March 1 to June 30, 2020 (during-COVID), respectively.

## Materials and methods

Study setting and study duration

This was a retrospective cross-sectional study conducted at the Aga Khan University Hospital (AKUH), in Karachi, Pakistan. AKUH is a Joint Commission International Accredited (JCIA) tertiary care teaching hospital, that provides services to a diverse population of patients presenting from the entire country and beyond.

We performed a comparison of two groups of patients who presented to the hospital with ischemic heart disease. The first group consisted of patients who presented to the hospital during March-June 2019, whereas, the second group of patients presented at a similar timeline during the COVID-19 pandemic (March-June 2020). Although March 23, 2020 marked the date when complete lockdown measures were instituted, we anticipated the reduction in volumes to have predated this (in early March), and hence collected complete data from March 1 to June 30 of each year. Admission date within these time frames was considered as the primary inclusion point.

Strategy for sample selection and data collection

Exhaustive data were collected on all adult patients (>18 years) who presented to the hospital during the aforementioned timelines with a hospital admission of ischemic heart disease. Inclusion criteria included all adult patients who were diagnosed with coronary atherosclerotic disease (stable) and the following components of the ACS: ST-elevation MI (STEMI), non-ST elevation MI (NSTEMI) and unstable angina (UA). With respect to the exclusion criteria, only patients who presented with these as their primary diagnosis were included, and patients with ACS as a secondary diagnosis were excluded. Cases with missingness of the requisite data and day-care oncology patients were excluded from the study. Consecutive sampling was used to include every patient who met the inclusion criteria during the study time period.

Data were procured retrospectively using physical and electronic chart review and health information management systems (HIMS). To ensure the accuracy and reliability of the vast dataset, two senior members of the research team supervised the data collection and validation process (5.69% records, n=58). Information and data points recorded included demographics, geographical location, clinical characteristics, management outcomes and pertinent admission and discharge details.

Data management protocol and ethical considerations

No direct or indirect contact was made with the patient or their family members. In order to duly protect the confidentiality of the subjects, all identifiers were removed subsequent to the data collection process using a unique 32-digit de-identified code. Only the coded data were shared with the full research team for data review and statistical analysis. This study was dually approved by both the Ethical Review Committee (Code: 2020-5246-11555), which serves as the Institutional Review Board at AKUH and by the Chief Medical Officer. No participant, study investigator or member of the research team had any additional benefits or risks, conflict of interest and funding disclosure to report.

Statistical analysis plan

Analysis of the data was done using the Statistical Package of Social Sciences (SPSS Version 26, IBM Corp., Armonk, NY) and STATA Version 16 (StataCorp, College Station, TX). For categorical variables, we reported frequencies and percentages (n, %), and measures of central tendency and variance (mean, median standard deviation and IQR) were used for the continuous variables. Group differences for continuous variables were evaluated using student t-test or Mann-Whitney U test. The chi-squared test or Fisher test was used for categorical variables. Values of p less than 0.05 were considered statistically significant. P-value trend calculation and graphical visualization were done using STATA.

## Results

Baseline characteristics of all patients

We collected data on a total of 1019 patients who met the inclusion and exclusion criteria, consisting of 706 (69.3%) and 313 (30.7%) patients in the pre-COVID and COVID eras, respectively. The two groups (Table 1) were quite similar in terms of age (62.4 ± 11.6 vs 62.4 ± 11.7, p=0.998), gender (males: 69.8% vs 71.2%, p=0.710) and known comorbidities such as diabetes mellitus (p=0.984), hypertension (p=0.386), dyslipidemia (p=0.964) and chronic kidney disease (p=0.242). Comparable proportions of ex-smokers were observed in both groups (24.2% vs 29.4%, p=0.088), however, there was a higher proportion of current smokers in the COVID era (13.9% vs 19.8%, p=0.019). All patients in the COVID era were screened for COVID-19 before admission. Only three patients were positive for COVID-19 swab reverse transcription-polymerase chain reaction (RT-PCR), however, mortality was not observed in any of these patients.

**Table 1:** Overall comparison of patient demographics, known comorbidities and admission rates in the pre-COVID and during-COVID eras, n=1019 *AKUH is located/centered in District East of Karachi. There are seven geographically divided administrative districts in the metropolitan city. This information was available for a subset of patients only; we have provided the locations of all patients for which it was procured. ^†^Independent t-test applied for continuous variables and the chi-squared test for categorical variables to assess the group differences. ^a^Fisher's exact test was used to assess the group differences.

Variable	Pre-COVID	During COVID	p-Value^†^
	March-June 2019	March-June 2020	
	n=706 (69.3%)	n=313 (30.7%)	
Age in years (mean ± SD)	62.4 ± 11.6	62.4 ± 11.7	0.998
Gender, n(%)			0.71
Male	493 (69.8)	223 (71.2)	
Female	213 (30.2)	90 (28.8)	
Known comorbidities, n (%)			
Diabetes mellitus	392 (55.5)	174 (55.6)	0.984
Hypertension	538 (76.2)	230 (73.5)	0.386
Ever smoker	171 (24.2)	92 (29.4)	0.088
Current smoker	98 (13.9)	62 (19.8)	0.019
Dyslipidemia	85 (12.0)	38 (12.1)	0.964
Chronic kidney disease	114 (16.1)	60 (19.2)	0.242
Reported geographic regions, n (%)	[n=538]*	[n=220]*	0.247^a^
District East, Karachi*	116 (21.6)	55 (25.0)	
Other districts, Karachi	269 (50.0)	107 (48.6)	
Outside Karachi (city)	79 (14.7)	34 (15.5)	
Outside Sindh (province)	64 (11.9)	24 (10.9)	
Outside Pakistan (country)	10 (1.9)	0 (0.0)	
Admission source, n (%)			<0.001
Emergency room (ER)	374 (53.0)	205 (65.5)	
Non-ER	332 (47.0)	108 (34.5)	
Time of admission, n (%)			0.221
Day (8 am-8 pm)	480 (68.0)	200 (63.9)	
Night (8 pm-8 am)	226 (32.0)	113 (36.1)	
Day of admission, n (%)			0.58
Weekday (Monday-Friday)	538 (76.2)	233 (74.4)	
Weekend (Saturday-Sunday)	168 (23.8)	80 (25.6)	
Month of admission, n (%)			<0.001
March	205 (29.0)	139 (44.4)	
April	203 (28.8)	61 (19.5)	
May	152 (21.5)	67 (21.4)	
June	146 (20.7)	46 (14.7)	

Admission and presentation

Most of the patients in both groups presented from within the city (71.6% vs 73.6%); there was an inverse relationship observed between admissions and distance from the facility. During the COVID era, although a higher proportion of patients presented from a different city within our province (14.7 vs 15.5%), fewer patients presented from a different city outside of our province (11.9 vs 10.9%), and no patients from outside of Pakistan could report to the facility (1.9 vs 0.0%). The two groups were similar in the time (p=0.221) and day of presentation (p=0.580) with higher numbers presenting in the day and on weekdays in both groups (Table 1).

An increasing trend of admissions from the ER was observed during the COVID era (53.0% vs 65.5%) showing that the admission source was significant (p<0.001). Overall admission rates by month declined significantly in the COVID era. During the pre-COVID era, a similar distribution of patients was observed across the individual months (29.0% vs 28.8% vs 21.5% vs 20.7%) compared to a sharp drop in the COVID era (44.4% vs 19.5% vs 21.4% vs 14.7%) in the months of March, April, May and June, respectively (p<0.001).

Differences in clinical characteristics, management and outcomes

We noticed a statistically significant increase in the proportion of overall ACS admissions (61.6% vs 76.7%, p<0.001) which corresponded to a significant decrease in the proportion of stable angina (38.4% vs 23.3%, p<0.001). Amongst the ACS patients, NSTEMI was showed increasing trends (38.1% vs 56.2%, p<0.001) with corresponding decreases in the proportions of unstable angina (p=0.025). Nevertheless, no change in STEMI cases was observed (13.0% vs 14.4%, p=0.561). Median (IQR) duration of stay, in hours, showed a slight increase from 70.1 hours (20.8-158.2) to 71.8 hours (38.4-142.7), which did not attain statistical significance (p=0.675). Overall ACS mortality and component-stratified mortality did not change significantly and remained static in the COVID era compared to the pre-COVID era (p=0.852). Varying trends in discharge disposition were observed; leave against medical advice (LAMA) and discharged for home showed decreases and discharged on request showed an increase, but these trends were unable to attain statistical significance.

A number of angiographies, angioplasties (PCI) and CABG procedures were plummeted significantly in the COVID era. However, the proportion of each procedure remained quite similar except for that of angiographies (Table 2), which showed a statistically significant drop (59.9% vs 49.2%, p=0.002).

**Table 2:** Clinical characteristics, short-term outcomes and management stratagem of patients presenting in the Pre-COVID and During-COVID eras, n=1019 ^†^Median (IQR); P-value computed by applying Mann-Whitney U Test.

Variable	Pre-COVID	During COVID	p-value
	March-June 2019	March-June 2020	
	n=706 (69.3%)	n=313 (30.7%)	
Disease presentation, n (%)			
Acute coronary syndrome	435 (61.6)	240 (76.7)	<0.001
STEMI	92 (13.0)	45 (14.4)	0.561
NSTEMI	269 (38.1)	176 (56.2)	<0.001
Unstable angina	74 (10.5)	19 (6.1)	0.025
Stable angina	271 (38.4)	73 (23.3)	<0.001
Duration of the stay [median (IQR)]^†^			
In hours	70.1 (28.0-158.2)	71.8 (38.4-142.7)	0.675^†^
In days	3.0 (1.0-6.0)	3.0 (2.0-6.0)	0.562^†^
Discharge disposition, n (%)			
Sent home	599 (84.8)	254 (81.2)	0.126
LAMA	48 (6.8)	18 (5.8)	
Discharge on request	32 (4.5)	23 (7.3)	
Expired	27 (3.8)	18 (5.8)	
Mortality, n (%)			
Total	27 (3.8)	18 (5.8)	0.852
Acute coronary syndrome	24 (5.5)	17 (7.1)	0.167
STEMI	6 (6.5)	5 (11.1)	0.353
NSTEMI	18 (6.7)	12 (6.8)	0.958
Unstable angina	0 (0.0)	0 (0.0)	NA
Stable angina	3 (1.1)	1 (1.4)	0.167
Management, n (%)			
Angiography	423 (59.9)	154 (49.2)	0.002
PCI/angioplasty	195 (27.6)	93 (29.7)	0.498
CABG	176 (24.9)	70 (22.4)	0.428
Medical treatment only	338 (47.9)	151 (48.2)	0.946

Key trends in ACS admission, discharge, presentation and management data

**Figure 1:**
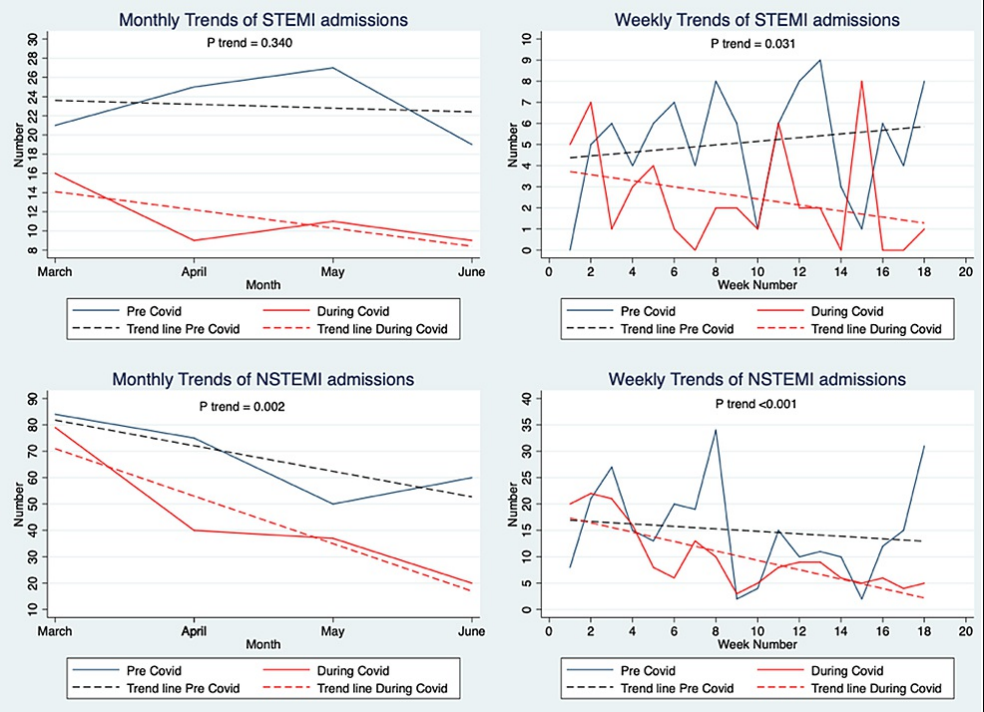
Monthly and weekly patterns in disease presentation of ACS patients in the pre-COVID and during-COVID eras

**Figure 2:**
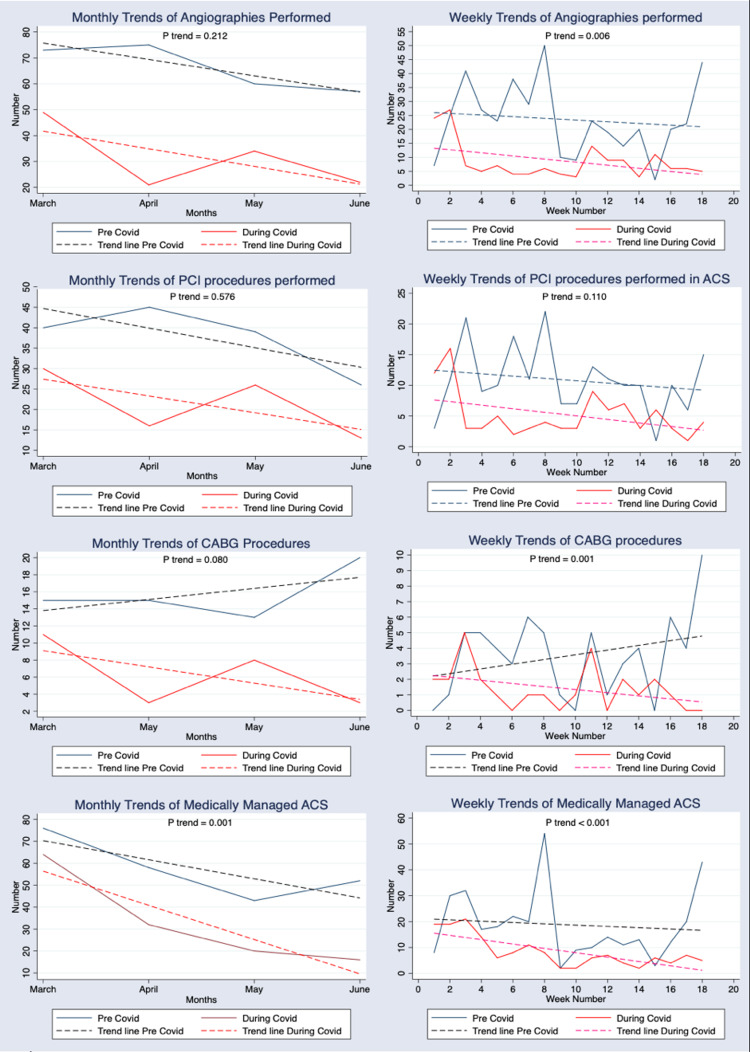
Monthly and weekly patterns in the management of ACS patients in the pre-COVID and during-COVID eras

Since the proportion of ACS presentations and the overall monthly distribution showed the highest levels of statistically significant differences, we carried out further trend analysis to investigate time-dependent patterns in variables within the two study periods (Table 3 and Figures 1-2).

**Table 3:** Monthly trends in patient descriptors for acute coronary syndrome in the pre-COVID and during-COVID eras *ACS admissions (%) = (total ACS admissions/all admissions *100). **AKUH is located/centered in District East of Karachi. There are seven geographically divided administrative districts in the metropolitan city. This information was available for a subset of patients only; we have provided the locations of all patients for which it was procured. ^¥^Monthly total is the sum of count for the same month in 2019 and 2020. n (row %) indicates (variable count in X month of 2020/variable count in X month in 2019 plus 2020 *100). ^†^P_trend_ is computed by using the STATA p-trend command.

Variable n(%)	March	March	April	April	May	May	June	June	p_trend_^†^
	Total^¥^	2020	Total^¥^	2020	Total^¥^	2020	Total^¥^	2020	
All admissions (n=20,318)	5828	2548 (43.7)	4917	1717 (34.9)	4855	1720 (35.4)	4718	1750 (37.1)	0.001
ACS admissions (n=675)	234	104 (44.4)	169	51 (30.2)	144	53 (36.8)	128	32 (25.0)	
ACS admissions (%)*	4	4.1	3.4	3	3	3.1	2.7	1.8	
Geographic region									<0.001
District East, Karachi**	51	24 (47.1)	34	9 (26.5)	18	9 (50.0)	24	7 (29.2)	
Other districts	85	33 (38.8)	66	23 (34.8)	60	22 (36.7)	42	7 (16.7)	
Outside Karachi	98	47 (48.0)	69	19 (27.5)	66	22 (33.3)	62	18 (29.0)	
Time of admission									0.038
Day (8 am-8 pm)	132	61 (46.2)	89	22 (24.7)	84	33 (39.3)	75	21 (28.0)	
Night (8 pm-8 am)	102	43 (42.2)	80	29 (36.3)	60	20 (33.3)	53	11 (20.8)	
Day of Admission									0.069
Weekday (Mon-Fri)	177	77 (43.5)	115	33 (28.7)	117	39 (33.3)	73	24 (32.9)	
Weekend (Sat-Sun)	57	27 (47.4)	54	18 (33.3)	27	14 (51.9)	55	8 (14.5)	
Disease presentation									
STEMI	37	16 (43.2)	34	9 (26.5)	38	11 (28.9)	28	9 (32.1)	0.34
NSTEMI	163	79 (48.5)	115	40 (34.8)	87	37 (42.5)	80	20 (25.0)	0.002
Unstable angina	34	9 (26.5)	20	2 (10.0)	19	5 (26.3)	20	3 (15.0)	0.493
Management									
Angiography	122	49 (40.2)	96	21 (21.9)	94	34 (36.2)	79	22 (27.8)	0.212
PCI/angioplasty	70	30 (42.9)	61	16 (26.2)	65	26 (40.0)	39	13 (33.3)	0.576
CABG	26	11 (42.3)	18	3 (16.7)	21	8 (38.1)	23	3 (13.0)	0.08
Medical only	140	64 (45.7)	90	32 (35.6)	63	20 (31.7)	68	16 (23.5)	0.001

In the monthly trend analysis, the overall geographic region showed a significant downward trend in intra-district, intra-city and outside city volumes (p<0.001). Time of admission also showed a significant trend (p=0.038), with lesser patients presenting over the night shift as the months progressed in the COVID era. This was not seen in the pre-COVID era. Differentiation between weekday and weekend admission did not show any significance in the trend (p=0.069). NSTEMI admissions and medical management of ACS admissions showed significant trends over the four months in the two eras (p=0.002 and p=0.001, respectively).

We also conducted weekly (pair of two weeks) trend analysis to explore any further significant trends that may have been missed due to assimilation of data by month. Although angioplasty procedures showed a non-significant downward trend across the months (p=0.110), many other variables did present significant downward trends. These included admissions (p=0.001), STEMIs (p=0.031), NSTEMIs (p<0.001), angiography procedures (p=0.006), CABG procedures (p=0.001) and medical management (p<0.001).

## Discussion

This retrospective study reveals three main findings: a significant overall decline in patients reporting with IHD (both ACS and elective admissions for stable CAD); a relative decrease in procedures (both diagnostic and therapeutic); and a statistically significant downward trend (month-wise) in the geographical area of presentation, time of admission and NSTEMI presentations which also correlated with Pakistan’s peak surges.

The overall decline in admissions

Our data appreciate the changes in the volume of admissions for ACS during the pandemic in the city of Karachi across the months March through till June in 2020 and the corresponding time period in 2019. A significant decrease in COVID era ACS admissions has been explained in the existing literature by an apprehension of healthcare settings due to possible SARS-CoV-2 infection and an emphasis on the management of COVID-related cases in hospitals [9,11-13]. Of further considerable note and attention, is the statistically significant reduction in the admission of non-emergency patients for ACS as compared to emergency cases. Studies have reported a decrease in the incidence of elective procedures due to indefinite cancellations of the same by hospitals [14,15].

In line with these global findings, our results demonstrate that while the ostensible decrease in STEMI admissions was insignificant, there is a noteworthy and statistically significant change in NSTEMI admissions. A smaller decrease in STEMI admissions as compared to NSTEMI admissions has been corroborated by several other studies globally [9,12,16]. Despite a myriad of determinants, the difference may be attributed to the severity of manifested symptoms of STEMI, requiring urgent in-hospital management, as compared to NSTEMI, which a patient may be able to endure at home [9]. Our study resonates with the global perspective that patients were not arriving at the hospital unless professional intervention was absolutely imperative.

Plummeting numbers of therapeutic and diagnostic procedures - relation to a global overview

Although it is felt conspicuously, the holistic adverse impact of the pandemic on healthcare systems is still relatively undocumented, if at all, in LMICs like Pakistan. Studies have shown considerable effects on both elective cardiac procedures performed globally [2] and emergency coronary procedures in Europe and the United States [9,17]. Diagnostic angiographies at our institution showed a reduction of 63.6%, with an almost 50% decrease within each month of pre- and during- COVID period. Similar trends were seen in England, where the decline in angiographies was estimated to be 60% [9]. In addition, decreased STEMI activations also led to a 34% reduction in angiography volumes in the US during COVID-prevalent months of March and April 2020 [16].

The number of revascularization PCI and CABG procedures dropped by more than half during the same time period in the COVID era. An overall reduction in PCI procedures was also noted worldwide, with a national survey in the United Kingdom showing a 43% decrease in PCI volumes by the end of April 2020. Although similar trends were seen in the US (20% reduction from 12 sites) [16], our study showed a greater decline of 52.3% in PCI procedures. The trend in CABG surgeries has also shown a parallel trajectory (>50% decrease) which reverberates with studies showing an almost negligible inflow of patients during the peak spread of COVID-19 in March and April 2020 [9]. A study conducted at 67 North American adult cardiac surgery institutions reported a 45% decline in cardiac surgery case volumes, highlighting similar decreasing trends for both high- and low-burden areas [18], meanwhile, the UK also showed a substantial reduction in their CABG cases of 80% within the National Health Services hospitals [9].

Relation of trends to national perspectives

These data correlate well with the timeline of the COVID-19 pandemic as well as the governments’ reactionary policies to combat it. The first two COVID-19 cases in Pakistan were confirmed on February 26, 2020 [19]. Since then, infections had initially increased exponentially, peaking in approximately mid-June, with a daily maximum of 6,825 new infections on June 13th, 2020 [20]. COVID-19 related deaths followed a similar trend, with a peak in the number of deaths in mid-June [20]. The Institute for Health Metrics and Evaluation (IHME) also estimated a peak in early June, with up to 95,000 daily infections [21].

With the start of the pandemic, national and provincial governments soon shut down educational institutions, religious centers, offices, restaurants and shopping centers; cancelled major sporting and entertainment events; and halted all air and rail travel [22]. In particular, hospitals were also ordered to shutter their outpatient departments (OPDs) and cancel non-emergent procedures and surgeries [23]. All of these culminated with several provincial governments (including Sindh, the province in which our tertiary care hospital is located) imposing province-wide lockdown, imposing stay-at-home orders and banning any and all non-essential travel outside one’s home [23]. Therefore, these trends can be described by one or more of the following reasons.

First, public perception and fear surrounding the COVID-19 pandemic may have led to patients being unwilling to leave their homes to seek medical attention when experiencing mild or moderate symptoms of ACS. It has been reported in studies relating to child health, stroke, inflammatory bowel disease and STEMI that fear of COVID-19 is a major reason for patients not presenting to a hospital or clinic when they experience symptoms of any kind [24-27]. This would explain the reduction in cases seen early in the month of March when lockdown measures had not yet taken effect. Intense media scrutiny, as well as general confusion surrounding the first few infections in the country, is likely to have led to patients voluntarily staying away from hospitals and clinics out of the fear of contracting the virus.

Second, restrictions in place due to quarantine and lockdown measures are likely to have had an effect on the ACS case volumes at our center. A complete shutdown of elective surgical procedures in the months late March through till June in hospitals across the country meant that patients with non-emergent cases simply did not get operated on [23]. This effect was further compounded by a hospital-wide shunting of resources towards the management of patients with COVID-19 infections at our center - with so many critical patients to care for, less administrative attention and importance was given to resuming elective surgical procedures.

Lastly, outpatient consultations (including cardiac OPDs) were temporarily restricted at the height of the lockdown as well. Delayed or missed patient consultations and a lack of routine screening are likely to have led to a decrease in PCI or CABG referrals. This is particularly evident in our data, wherein there was a statistically significant decline in the number of cases referred to surgery from “non-ER” - i.e., outpatient consulting clinics.

Strengths, limitations and future directions

To the best of our knowledge, this is the first large-scale data-driven study that endeavors to shed light on the consequences of the COVID-19 pandemic on non-COVID cardiovascular healthcare in Pakistan. LMICs may stand to derive tangible benefit from such studies, as they allow for the creation of future public health recommendations and guidelines which are derivative of contextual and regional data.

Our triphasic approach to sequential and methodological analysis plan shows data comparing overall volumes, monthly trends and weekly trends. This allows for both a birds-eye view of the entire course as well as greater granularity with time-specified data points. It is also interesting to note that these monthly and weekly trends correlate well with the onset of lockdown measures and the peak of cases in June 2020. Our data also adds two novel facets to the existing literature on the management of ACS patients during the pandemic. These include firstly, the declining trends in non-invasive medical treatment for ACS patients, and secondly, a statistically significant comparison of elective vs non-elective admissions during the same calendar months in the two eras.

Although the volumes of admissions and procedures captured during these time periods are substantial in number, an important limitation is that our data still stems from a single tertiary care center. This creates the possibility that the data are not generalizable across the country. However, AKUH is one of the largest cardiac centers in Pakistan with many reported admissions coming in from all over the country and beyond. We anticipate that our observed trends in admission volumes, procedures, patient presentations and outcomes would likely follow the general nationwide trends. That said, this limitation cannot be dismissed, which makes it imperative to use these data only as a pilot that paves the way for many larger multi-center studies. We also recognize that the nature of the data is short-term and does not predict the long-term patient outcomes or the lasting implications on the healthcare landscape. However, we hope that the headway made in this study garners a more robust understanding of the continuing sequelae of the pandemic’s adverse effects.

## Conclusions

Our study shows critical reductions in the number of ACS, therapeutic and diagnostic procedures during the COVID-19 pandemic in a developing country. In a country where resources and research are both scarce, it would be remiss to not make attempts at comprehending the short-term and long-term implications of this untreated ischemic heart disease. 

To begin with, this study should be used in conjunction with future studies evaluating the out-of-hospital cardiac arrests (OCHA) during this pandemic. Furthermore, there is an urgent need to delve into the reasons why expected health-seeking behaviour in emergent cases was undervalued by the community, even in the context of this pandemic. An in-depth understanding of this community phenomenon, in conjunction with hospital data, should be the guiding fundamentals of healthcare policy-making for the years to come.
